# Synthesis, Surface Nitriding and Characterization of Ti-Nb Modified 316L Stainless Steel Alloy Using Powder Metallurgy

**DOI:** 10.3390/ma14123270

**Published:** 2021-06-13

**Authors:** Sadaqat Ali, Muhammad Irfan, Usama Muhammad Niazi, Ahmad Majdi Abdul Rani, Imran Shah, Stanislaw Legutko, Saifur Rahman, Mohammed Jalalah, Mabkhoot A. Alsaiari, Adam Glowacz, Fahad Salem AlKahtani

**Affiliations:** 1 School of Mechanical & Manufacturing Engineering, National University of Sciences and Technology (NUST), H-12, Islamabad 44000, Pakistan; 2 Electrical Engineering Department, College of Engineering, Najran University Saudi Arabia, Najran 61441, Saudi Arabia; miditta@nu.edu.sa (M.I.); srrahman@nu.edu.sa (S.R.); fsalkahtani@nu.edu.sa (F.S.A.); 3 Mechanical Engineering Department, National University of Technology, Islamabad 44000, Pakistan; imranshahswabi@gmail.com; 4 Mechanical Engineering Department, National Skills University, Islamabad 44000, Pakistan; 5 Mechanical Engineering Department, Universiti Teknologi PETRONAS (UTP), Bandar Seri Iskandar 32610, Perak Darul Ridzuan, Malaysia; majdi@utp.edu.my; 6 Faculty of Mechanical Engineering, Poznan University of Technology, 3 Piotrowo str., 60-965 Poznan, Poland; 7 Promising Centre for Sensors and Electronic Devices (PCSED), Najran University Saudi Arabia, Najran 61441, Saudi Arabia; msjalalah@nu.edu.sa; 8 Empty qaurter research unit, Chemistry department, college of Science and art at Sharurah, Najran University Saudi Arabia, Najran 61441, Saudi Arabia; mabkhoot.alsaiari@gmail.com; 9 Department of Automatic Control and Robotics, Faculty of Electrical Engineering, Automatics, Computer Science and Biomedical Engineering, AGH University of Science and Technology, al. A. Mickiewicza 30, 30-059 Kraków, Poland; adglow@agh.edu.pl

**Keywords:** surface nitriding, sintering, 316L stainless steel, titanium, niobium, biomedical

## Abstract

The powder metallurgy (PM) technique has been widely used for producing different alloy compositions by the addition of suitable reinforcements. PM is also capable of producing desireable mechanical and physical properties of the material by varying process parameters. This research investigates the addition of titanium and niobium in a 316L stainless steel matrix for potential use in the biomedical field. The increase of sintering dwell time resulted in simultaneous sintering and surface nitriding of compositions, using nitrogen as the sintering atmosphere. The developed alloy compositions were characterized using OM, FESEM, XRD and XPS techniques for quantification of the surface nitride layer and the nitrogen absorbed during sintering. The corrosion resistance and cytotoxicity assessments of the developed compositions were carried out in artificial saliva solution and human oral fibroblast cell culture, respectively. The results indicated that the nitride layer produced during sintering increased the corrosion resistance of the alloy and the developed compositions are non-cytotoxic. This newly developed alloy composition and processing technique is expected to provide a low-cost solution to implant manufacturing.

## 1. Introduction

The use of austenitic 316L stainless steel (SS) as a biomaterial can be traced back to the distant past for producing implants and medical devices. It all started with development of medical implants for patients with advised surgeries including total hip replacement [1,2]. Subsequently, this material gained attention and is widely used in several biomedical applications including implantation and medical devices. This material shows adequate mechanical properties and biocompatibility at a reasonable cost [3,4,5,6]. The implants produced from this material are much cheaper as compared to other biomaterials available in the market including titanium, cobalt chromium and others [7,8,9,10]. The implants manufactured from this material have adequate strength to maintain their structure in high loading conditions and also possess ease of fabrication [11]. The ASTM International also recommends 316L stainless steel for producing implants and medical devices [12,13,14]. 316L stainless steel has a high chromium concentration and low carbon content which makes it an ideal material in chlorine bearing solutions [15]. The human saline closely resembles chlorine bearing solutions making it a suitable biomaterial for implantation [16].

The 316L stainless steel contains nickel, with a percentage of around 10–14%. The presence of nickel in the stainless steel matrix helps in maintaining austenitic structure of this material which is required for biomedical applications [17,18]. However, the presence of nickel on the other hand may cause allergic reactions in patients if this nickel is released into the human body [19,20]. Reports from patients indicate the presence of nickel in the human body, indicating that debris and metal ions have been released from the implant material. This can be due to inferior corrosion resistance and poor surface finish [21,22,23]. The reported diseases due to ionic leaching include genotoxic and mutagenic activities, skin diseases including eczematous rash, dermatitis and swelling [24,25,26]. The leaching of ions from the biomaterials demand surface modification along with improving the composition of this material for prospective use as a successful biomaterial.

Powder metallurgy is one of the promising processing methods to improve the alloy composition [18,27]. This research has attempted to further accelerate this technique for not only developing the alloy composition but also the surface nitride the layer of implant material with an aim to minimize the ionic leaching of nickel and other elements. This has been achieved by enhanced sintering dwell time under a nitrogen atmosphere. This enhanced dwell time diffused nitrogen into the stainless steel matrix resulting in the formation of strong nitrides of iron, chromium, nickel and others. To modify the composition of 316L stainless steel, titanium (Ti) and niobium (Nb) have also been added in the developed compositions to make this material more promising for implant manufacturing. Pure titanium is one of the biomaterials that has been used in implant manufacturing due to its inherent properties and exceptional corrosion resistance [28,29,30]. In recent times, niobium has also gained considerable attention due to its enhanced properties and potential use in biomedical applications [31,32,33]. The addition of these reinforcements has been selected so as to maintain the austenitic structure of the developed compositions. The sintering parameters have also been selected so as to create a surface nitride layer on the developed compositions.

## 2. Materials and Methods

The research started by taking 316L stainless steel as the base material with titanium and niobium as the reinforcements in different ratios. The composition of the as received 316L stainless steel powder from the supplier (Wuxi Eternal Bliss Alloy Casting & Forging Co., Ltd., Xishan District, Wuxi, Jiangsu, China) is presented in Table 1.

**Figure 1:**
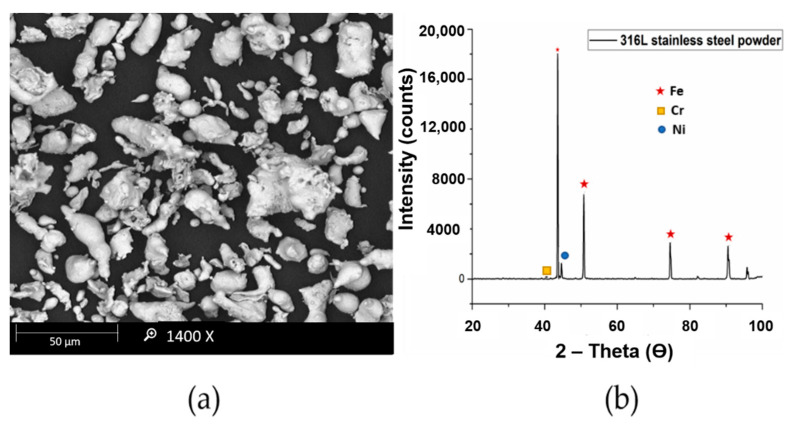
(**a**) SEM (**b**) XRD analysis of 316L stainless steel powder.

The scanning electron microscopy (SEM) and XRD analysis of 316L stainless steel powder is presented in Figure 1. The average particle size of the powder is about 15 μm with irregular shape.

**Figure 2:**
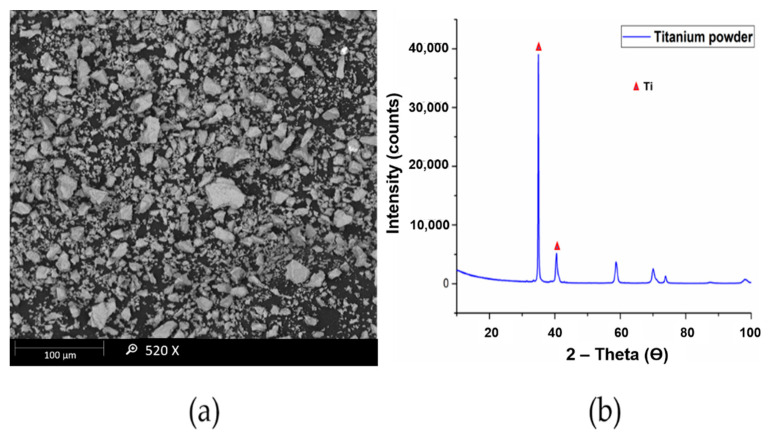
(**a**) SEM (**b**) XRD analysis of titanium powder.

**Figure 3:**
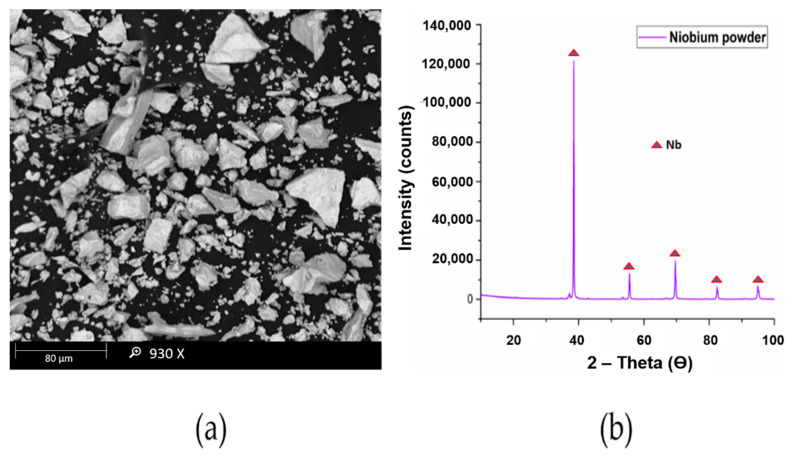
(**a**) SEM (**b**) XRD analysis of niobium powder.

The titanium powder used in this research had a particle size of 10 μm as observed through MASTERSIZER 2000 and was supplied by Chengdu Huarui Industrial Co. Ltd., Qingyang District, Chengdu, China. The niobium powder used in this research work was supplied by Chengdu Haoxuan Co. Ltd., Sichuan, China. The particle size of the powder was found to be 10 μm using MASTERSIZER 2000. The Scanning electron microscopy (SEM) and X-Ray diffraction (XRD) analysis of titanium and niobium powders are presented in Figure 2 and Figure 3, respectively.

In this study, four formulations were designed for the development of a modified stainless-steel alloy. The effect of each powder concentration on the resultant alloy system was studied in terms of sintered density, mechanical properties, corrosion resistance and in-vitro cytotoxicity assessment. Pure 316L stainless steel was taken as the first formulation whereas the next three formulations contained titanium and niobium admixed in a 316L stainless steel matrix. The details of these formulations have been presented in Table 2.

The titanium–niobium admixed 316L stainless steel formulations were prepared by mixing titanium, niobium and 316L stainless steel powders in their respective ratios. Before mixing, these powders were placed in vacuum oven (Lindberg/Blue M Digital Vacuum Oven, Thomas Scientific P.O. Box 99, Swedesboro, NJ, USA) under a temperature of 80 °C for 5 h to remove any moisture present in the powders. The powder combinations were prepared by mixing the corresponding ratios of each powder using turbula mixer.

Uniaxial cold compaction was carried out on all the four formulations of powders using a 3000 kN single action uniaxial hydraulic press. The powders were compacted at 800 MPa to achieve maximum green density. The obtained samples were of disc shape with 5 mm thickness and 30 mm diameter. The compacted samples were then pressureless sintered in a tube furnace. The sintering atmosphere was nitrogen, and the sintering parameters included a temperature of 1200 °C with a dwell time of 8 h. The sintered samples were then analyzed for densification, characterization, micro hardness, corrosion resistance and cytotoxicity assessment.

The green density of compacted samples indicates the compressibility of metal powder at a certain compaction pressure. The values of green densities for each sample were calculated via mass per unit volume. The sintered density of each sample was calculated using Archimedes’ principle via standard test method (ASTM B962-14) using an HR-150 AZ analytical balance (A&D Company, Limited, Tokyo, Japan).

The microstructure of sintered samples was observed through an optical microscope (Leica DM LM, Wetzlar, Germany). Variable pressure field emission scanning electron microscopy (FESEM) (VPFESEM Zeiss Supra 55VP, Oberkochen, Germany) was used to examine the sintered samples and investigate the microstructure of the sintered samples along with the elemental mapping.

The XRD analysis was performed for the sintered samples using an XRD (PAN analytical X’pert3, X’pert3, Powder and Empyrean, B.V, Lelyweg, Almelo, The Netherlands). An anode of Copper (Cu) K(alpha) with a wavelength of 1.5 Å was used in this study. A scan range of 10–90° with 1 °/min steps was used for all the samples at room temperature. The XRD analysis revealed the different compounds present in the matrix.

X-Ray Photoelectron Spectroscopy (XPS) (Thermo scientific, K-alpha, East Grinstead, UK) was employed to investigate the percentage of nitrogen and other elements present on the surface of the sintered sample.

In order to measure the tensile strength of the developed alloy systems, flat dumbbell shape tensile samples with a gauge section of 10 mm (length) × 2.5 mm (width) × ~ 5 mm (thickness) were cut from the disc shape sintered specimen via electro-discharge machining (EDM) according to the ASTM B925-08 (E8/E8M–13a) standard. The tensile testing was performed using a Shimadzu universal tensile testing machine. The strain rate was kept at 0.01 mm/min for all samples to avoid heat generation and sudden jerks.

The micro hardness was determined using a Vickers hardness tester (Leco LM 247AT, St Joseph, MI, USA). A force of 200 gf with 15 s dwell time was applied on all the compositions and at least five values were recorded for each sample from different locations on the test samples and the averaged values were calculated.

The weight loss approach was utilized to find the corrosion resistance of sintered samples. For this, an artificial saliva solution was prepared according to the literature [34,35], and sintered samples were immersed in the solution to investigate the weight loss. Before immersing, the weight of each sample was calculated. A time period of 28 days was given to the samples to remain in the solution. The samples were weighed again after cleaning and the weight loss was calculated accordingly.

The atomic absorption spectroscopy (AAS) was performed using GTA120 Graphite Tube Atomizer (Agilent Technologies, Stevens Creek Blvd, Santa Clara, CA, USA) to investigate and quantify the number of released ions in the artificial saliva solution after 28 days of immersion.

The sintered samples were tested for cytotoxicity using fibroblast cell line culture (NIH/3T3 ATCC^®^ CRL-1658). Dulbecco’s modified Eagle’s medium (DMEM) was utilized in the testing whereby, NIH/3T3 cells were expanded in the media. It contained 100 μg/mL of Pen/strep and 10 percent fetal bovine serum. An incubator was used to expand the cells at room temperature. The separation of the cells was carried out using trypsin–EDTA and the separated cells were then seeded on each sintered sample. A fluorescence plate reader was utilized to witness the absorbance over 3–4 h. An oxidation-reduction indicator changed the colour from blue (oxidized) to red (reduced) on the REDOX indicator, showing metabolic activity of the cells.

## 3. Results and Discussion

### 3.1. Measurement of Density

A green density of 6.5 g/cm^3^ was observed for pure 316L samples whereas, titanium-niobium modified 316L SS samples had lower green density values. A lower green density value can be attributed to the distribution of titanium and niobium particles in the SS matrix. Additionally, titanium is a low-density element as compared to SS resulting in lower green density of the overall sample compositions.

The sintered density was measured using Archimedes’ principle and was observed to be 7.575 g/cm^3^ for pure 316L SS samples. The relative density of pure 316L stainless steel samples was 95.88% of the theoretical density of 7.90 g/cm^3^ and was the highest among all the formulations developed.

The green and sintered density values have been presented in Table 3.

### 3.2. Microstructural Analysis and Elemental Mapping

**Figure 4:**
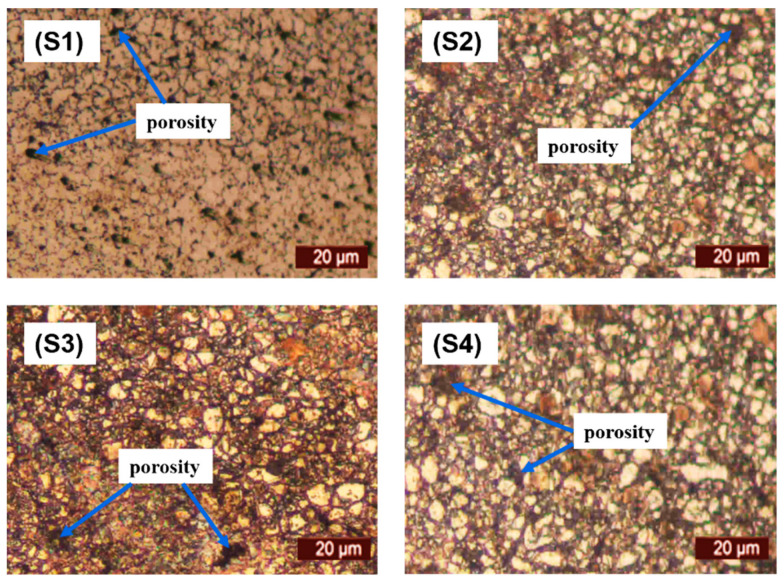
Microstructure of sintered samples (**S1**–**S4**).

The microstructural characterization of the sintered samples was carried out via optical microscopic observation. The micrographs viewed under microscope are depicted in Figure 4. It can be observed that pure 316L stainless steel has a dense structure with clear grain boundaries. Furthermore, the addition of titanium and niobium did not provide a barrier during the sintering process with significantly low porosity.

**Figure 5:**
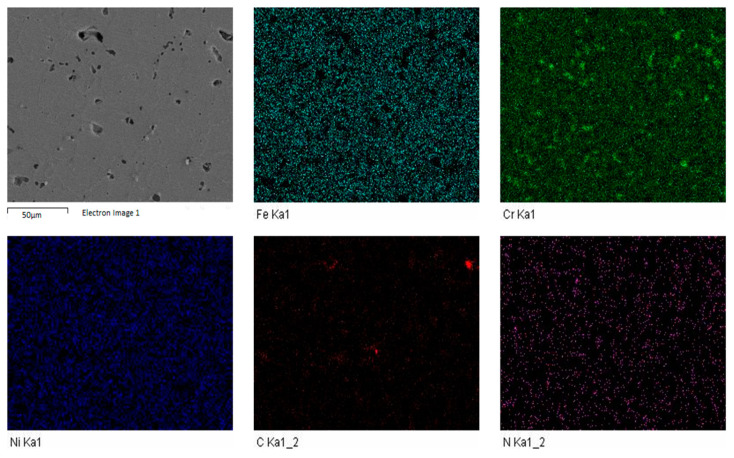
FESEM-EDS elemental mapping for sample S1.

**Figure 6:**
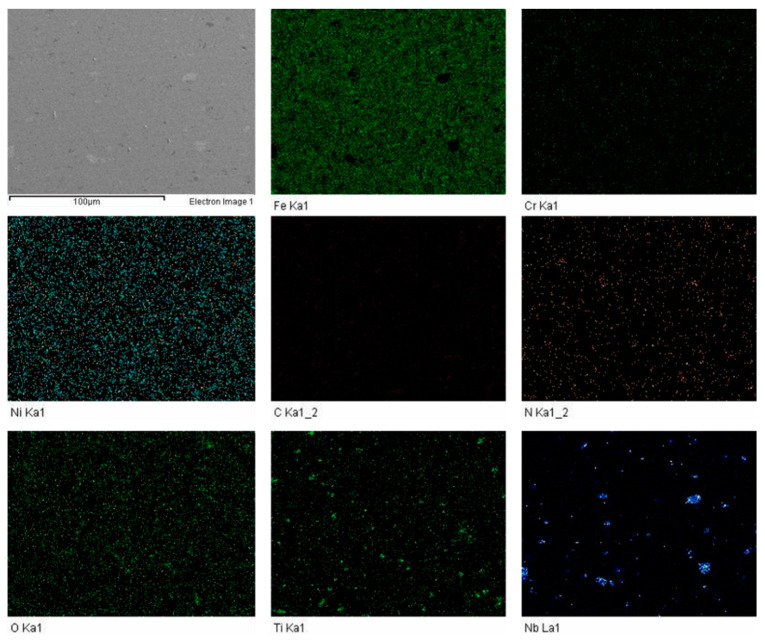
FESEM-EDS elemental mapping for sample S3.

The FESEM was carried out to investigate the influence of sintering parameters along with boron, titanium and niobium additions. The elemental mapping was carried out to discover the impact of enhanced sintering dwell time. It was determined that nitrogen was present in all the samples. The elemental mapping of samples S1 and S3 is shown in Figure 5 and Figure 6.

### 3.3. Surface Nitriding

The presence of the surface nitride layer was visualized using an optical microscope. The enhanced dwell time of 8 h not only diffused nitrogen into the matrix but was also able to develop a strong nitride layer onto surface of the sintered samples. This surface nitride layer contributed towards improved the mechanical and physical properties of the sintered samples.

**Figure 7:**
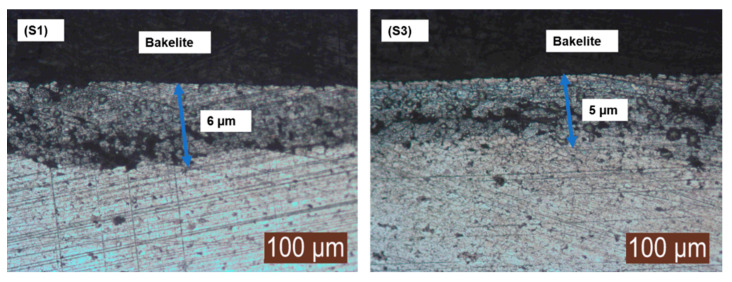
Surface nitride layer formation.

The nitride layer formation viewed under optical microscope of sample S1 and S3 is presented in Figure 7, supporting the statement of simultaneous sintering and surface nitriding using enhanced dwell time.

### 3.4. XRD Analysis

**Figure 8:**
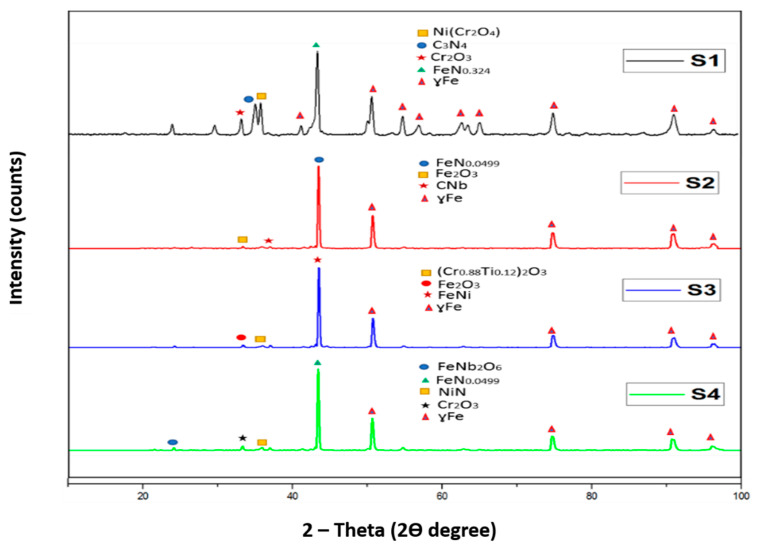
XRD analyses of sintered samples.

XRD analysis was carried out to examine the different compounds present in sintered samples. The XRD analyses are shown in Figure 8.

The XRD analysis demonstrates the austenitic structure (ɣ Fe) of all sample compositions. The formation of nitrides of different elements indicated that the nitrogen formed different compounds with elements of 316L stainless steel. The carbon and iron in sample S1 reacted with nitrogen forming their respective nitrides while for sample S2 it favoured the formation of iron nitride. A complete analysis of the different compounds formed is presented below:

The XRD analysis for the pure 316 L stainless steel sample indicated the formation of carbon nitride with a d-spacing of 2.51960 Å and iron nitride with a d-spacing of 2.07500 Å, along with nickel chromium oxide with a d-spacing of 2.49354 Å, and chromium oxide with a d-spacing of 2.66348 Å. The XRD pattern for sample S2 revealed the formation of iron nitride with a d-spacing of 2.08019 Å, iron oxide with a d-spacing of 2.68151 Å and niobium carbide with a d-spacing of 2.58249 Å. Sample S3 showed the presence of iron nickel with a d-spacing of 2.06932 Å, chromium titanium oxide with a d-spacing of 2.67259 Å and iron oxide with a d-spacing of 2.65941 Å. For sample S4, the XRD analysis demonstrated the presence of nickel nitride with a d-spacing of 2.49415 Å, iron niobium oxide with a d-spacing of 3.65506 Å, iron nitride with a d-spacing of 2.08019 Å and chromium oxide with a d-spacing of 2.66591 Å.

### 3.5. XPS Analysis

**Figure 9:**
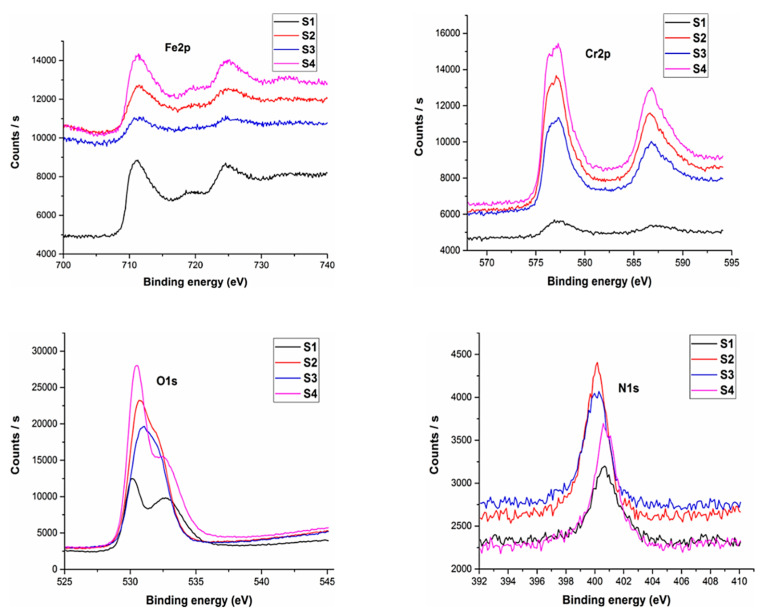
XPS analysis of sintered samples.

The XPS analysis of the sintered samples has been shown in Figure 9. XPS analysis was conducted to ascertain the various elements present on the sample surface.

The analysis revealed the existence of nitrogen for all the sample compositions. Oxygen was present with the greatest percentage followed by iron and chromium. An inherent property of austenitic stainless steel is the formation of chromium and iron oxides. These oxide layers form a passive film onto the sample surface.

The results indicated that 2.82% nitrogen was present for pure 316L stainless steel samples whereas a maximum amount of 3.67% was observed for sample S2, which was the maximum among all developed compositions. The percentage of nitrogen for sample S3 was 2.7% and 3.33% for sample S4 was observed. This indicates that the sintering parameters developed a nitride layer along with chromium and iron oxide layers that serve as a coating thereby helping to prevent the leaching of metal ions which is one of the main issues with 316L stainless steel.

### 3.6. Tensile Testing

The tensile strength of all the formulations was calculated using a tensile testing machine. The tensile load was applied at a constant rate of 0.01 mm/min. The ultimate tensile strength (UTS), ductility (% elongation) and Young’s modulus (E) was calculated from the stress–strain curve obtained from the tensile testing.

The tensile testing was carried out according to ASTM standards and the results for the testing have been tabulated in Table 4. The results indicate that the ultimate tensile strength (UTS) for pure 316L stainless steel samples was found to be 572.5 MPa, which was the maximum among all the formulations studied in this research. The higher UTS value may be attributed to the increased dwell time which helped in increasing the UTS of the samples. The percentage elongation for the sample was also the highest among all the samples and was calculated to be 25.08%. The tensile strength of Ti-Nb 316L SS formulations indicate that it has a deteriorating effect on the tensile strength of 316L stainless steel. The results also indicate that UTSs of relatively increased niobium content alloys have better tensile strength results as compared to titanium addition. A maximum of 431.76 MPa of UTS was observed for the S2 sample and the lowest was found for the S3 sample.

### 3.7. Microhardness

The Vickers hardness testing method was employed to find out the microhardness of the sintered samples. The microhardness values are shown in Table 5. A microhardness of 235 HV was noticed for pure 316L stainless steel. The microhardness of titanium-niobium modified 316L stainless steel formulations showed an increase in the microhardness of the samples. A maximum microhardness of 350 HV was observed for S4 sample.

### 3.8. Corrosion Resistance

The weight loss method was utilized to find the corrosion resistance of all the formulations. The weight loss measurements before and after immersion have been tabulated in Table 6 along with the weight loss that took place in 28 days.

It can be noticed that negligible weight loss is present for all the formulations. A weight loss of 0.05 g was found for pure 316L SS samples and was the highest weight loss. Sample S2 showed a weight loss of 0.02 g which showed the highest resistance to corrosion. The results of weight loss testing indicate that leaching of metal ions was very little from the samples due to the presence of strong layers on the sample surface, thus addressing the issue of leaching of metal ions.

### 3.9. Atomic Absorption Spectroscopy

AAS was performed to investigate and quantify the number of released ions in the artificial saliva solution after 28 days of immersion. A healthy man of approximately 70 kg possesses 10 mg of nickel in his body. This corresponds to 0.1 ppm of nickel concentration which is far less than the value of 30 ppm at which cytotoxicity occurs [36]. The concentrations of nickel released for all the samples are significantly less than that present in the human body. The results indicate the material composition, and their processing techniques are well suitable for developing biomaterials with minimal leaching of metals ions. The concentration of Fe, Cr and Ni ions released from sintered samples has been shown in Table 7.

### 3.10. In Vitro Cytotoxicity

NIH3T3 ATCC^®^ CRL-1658 (fibroblast cell line) was cultured on the sintered samples. The cell proliferation was assessed on day 3 for all the samples. The cell viability of the liquid chemicals was assessed using microplate reader absorbance graph analysis and the results were then compared with the control.

**Figure 10:**
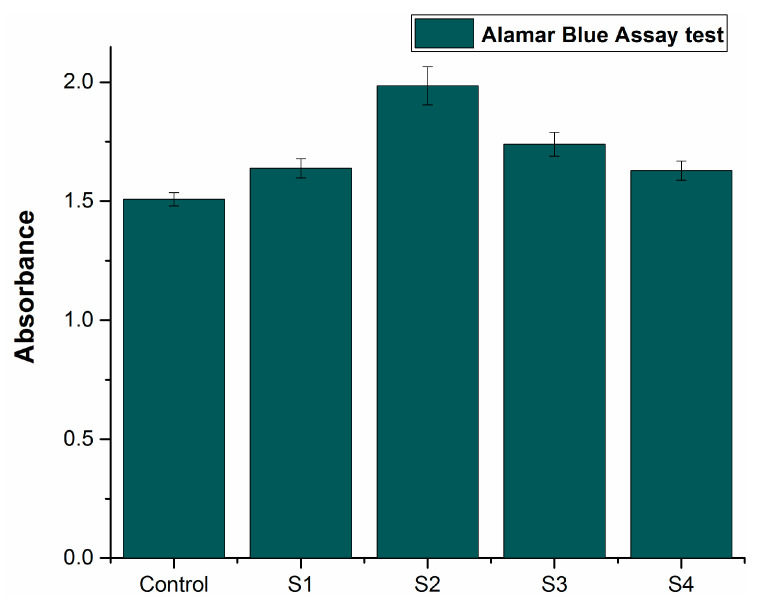
Comparison of control with samples.

Figure 10 shows the comparative results, indicating that the proliferation increased with time for deep eutectic solvents, in contrast with the control. The increased absorbance rate suggests increased cell proliferation. All the alloy compositions demonstrated enhanced absorbance as compared to the control. Sample S2 showed the highest cell proliferation, indicating the most antibacterial properties among all the compositions. These results indicate that all the developed formulations in this research work are compatible with living cells and non-cytotoxic in nature.

## 4. Conclusions

It can be concluded from these research findings that:

1. Simultaneous sintering and surface nitriding is possible by enhanced sintering dwell time.
2. The optimal sintering parameters enhance the mechanical, physical and surface properties. A sintering temperature of 1200 °C with an 8 h dwell time helps in developing a surface nitride layer along with diffusion of nitrogen into the matrix.
3. Nitrogen atmosphere is useful in nitride layer formation during sintering which minimizes the leaching of metal ions from the material.
4. The surface properties of pure and modified 316L stainless steel alloy have been enhanced for potential use in biomedical implant applications.
5. Although the relative density was decreased from 95.88% for pure 316L stainless steel sample to 90.71% for the alloy in S3, it is still in the acceptable range for producing implants. The microstructure of all the samples revealed significantly low porosity.
6. There is an appreciable increase in the microhardness of the sintered samples from 235 HV for pure 316L stainless steel samples to 350 HV for alloy containing 1.5% Ti and 0.5% Nb. This indicates that the addition of these elements considerably increased the microhardness of the sintered samples.
7. The results of corrosion resistance testing revealed that all the samples showed good corrosion resistance with minimal weight loss for all the samples. This indicates that the surface nitride layer formed during sintering served as a coating that helped in minimizing the leaching of metal ions from the samples as compared to the surfaces without the nitride layer [37].
8. The in vitro cytotoxicity results indicate that all the formulations developed in this research work are non-cytotoxic and are suitable for use in implant manufacturing.

## Figures and Tables

**Table 1:** Composition of 316L stainless steel powder.

Element	Fe	C	Cr	Ni	Mn	O	Mo	S	Si
Wt.%	Balance	0.028	17.04	12.01	1.5	0.068	2.4	0.008	0.9

**Table 2:** Alloy compositions used in this study.

S. No	Composition	Alloy Name
1	Pure 316L stainless steel	S1
2	316L SS + 0.5 Ti + 1.5 Nb	S2
3	316L SS + 1.0 Ti + 1.0 Nb	S3
4	316L SS + 1.5 Ti + 0.5 Nb	S4

**Table 3:** Green and sintered densities of all compositions.

Composition	Theoretical Density (g/cm^3^)	Green Density (g/cm^3^)	Sintered Density (g/cm^3^)	Relative Density (%)
Pure 316L stainless steel	7.90	6.5	7.575	95.88
316L SS + 0.5 Ti + 1.5 Nb	7.886	6.192	7.197	91.26
316L SS + 1.0 Ti + 1.0 Nb	7.864	6.196	7.134	90.71
316L SS + 1.5 Ti + 0.5 Nb	7.842	6.108	7.126	90.86

**Table 4:** Tensile test results of sintered samples.

Composition	Ultimate Tensile Strength (MPa)	Percentage Elongation (%)
Pure 316L stainless steel	572.5	25.08
316L SS + 0.5 Ti + 1.5 Nb	431.76	13.52
316L SS + 1.0 Ti + 1.0 Nb	409.34	14.91
316L SS + 1.5 Ti + 0.5 Nb	413.67	11.90

**Table 5:** Microhardness of sintered samples.

Composition	Microhardness
Pure 316L stainless steel	235 HV
316L SS + 0.5 Ti + 1.5 Nb	327 HV
316L SS + 1.0 Ti + 1.0 Nb	338 HV
316L SS + 1.5 Ti + 0.5 Nb	350 HV

**Table 6:** Weight loss measurements in artificial saliva solution.

Composition	Weight Before Immersion (g)	Weight After Immersion (g)	Weight Loss (g)
Pure 316L stainless steel	17.310	17.260	0.05
316L SS + 0.5 Ti + 1.5 Nb	18.140	18.120	0.02
316L SS + 1.0 Ti + 1.0 Nb	18.180	18.140	0.04
316L SS + 1.5 Ti + 0.5 Nb	18.140	18.110	0.03

**Table 7:** Concentration of ions released.

Composition	Elements Concentration in ppm		
	Fe	Cr	Ni
Pure 316L stainless steel	0.005	0.001	0.081
316L SS + 0.5 Ti + 1.5 Nb	0.001	0.000	0.065
316L SS + 1.0 Ti + 1.0 Nb	0.007	0.003	0.075
316L SS + 1.5 Ti + 0.5 Nb	0.004	0.001	0.050

## Data Availability

Not applicable.
